# Online Multivariate Anomaly Detection and Localization for High-Dimensional Settings

**DOI:** 10.3390/s22218264

**Published:** 2022-10-28

**Authors:** Mahsa Mozaffari, Keval Doshi, Yasin Yilmaz

**Affiliations:** Electrical Engineering Department, University of South Florida, Tampa, FL 33620, USA

**Keywords:** anomaly detection, change detection, anomaly localization, nonparametric methods, sequential analysis

## Abstract

This paper considers the real-time detection of abrupt and persistent anomalies in high-dimensional data streams. The goal is to detect anomalies quickly and accurately so that the appropriate countermeasures could be taken in time before the system possibly gets harmed. We propose a sequential and multivariate anomaly detection method that scales well to high-dimensional datasets. The proposed method follows a nonparametric, i.e., data-driven, and semi-supervised approach, i.e., trains only on nominal data. Thus, it is applicable to a wide range of applications and data types. Thanks to its multivariate nature, it can quickly and accurately detect challenging anomalies, such as changes in the correlation structure. Its asymptotic optimality and computational complexity are comprehensively analyzed. In conjunction with the detection method, an effective technique for localizing the anomalous data dimensions is also proposed. The practical use of proposed algorithms are demonstrated using synthetic and real data, and in variety of applications including seizure detection, DDoS attack detection, and video surveillance.

## 1. Introduction

Anomaly detection is an important problem dealing with the detection of abnormal data patterns [1]. It has applications in a variety of different domains, such as automatic video surveillance [2], cybersecurity [3], medical health care [4], and quality control. The importance of anomaly detection lies in the fact that an anomaly in the observation data may be a sign of an unwanted event such as failure and malicious activity. in the underlying system. Therefore, accurate detection of such data patterns will allow proper countermeasures to be taken by the domain specialist to counteract any possible harm. The advances in various technologies such as Internet-of-Things (IoT) devices and sensors, and wireless communications, have enabled the real-time monitoring of systems for detecting events of interest. In many modern and complex systems, such as IoT networks, network-wide traffic monitoring systems, environmental monitoring systems, massive amounts of heterogeneous data are generated, which require real-time processing for timely detection of anomalous events. As an example, automated vehicles or advanced driver-assistance systems today are equipped with modules comprising a large number of sensors and actuators for control and safety purposes. Due to the catastrophic consequences of any fault in perceiving the environment or failure in a component of the system, as well as being compromised by hackers, it is crucial to preserve the robustness of the vehicle. To this end, the high-dimensional measurements from sensors need to be monitored and analyzed in real-time to detect anomalies such as sudden increase of speed, abnormal petrol consumption, anomalies in radar sensors and camera sensing [5]. Accurate and light-weight anomaly detection methods that can scale well to large systems are needed to be able to address such big data challenges in real-time.

Anomaly detection methods on univariate data streams have been studied thoroughly in the literature. However, little work has been done on multivariate anomaly detection, which has the potential to achieve quicker and more accurate detection than univariate anomaly detection by capturing more anomaly evidence in the interactions between system dimensions. Statistical approaches to anomaly detection assume anomaly to be a change in the probability distribution of the observations, such as a change in the mean, variance, or correlation structure between the data-streams. One important application for detecting changes in the correlation structures is finance, where the correlation structures between high-dimensional processes modeling the exchange rates and market indexes are important for the right choice of asset allocation in portfolio [6]. Furthermore, in social networks, it is important to detect abrupt changes in interactions between the nodes; and in communication networks, it is of interest to detect highly correlated traffic in a network [7]. Distributed Denial of Service (DDoS) attacks to power grid through synchronous switching on/off of high-wattage IoT devices is another example where anomaly is manifested in correlations [8]. Detection of change in correlation structure requires the joint monitoring and multivariate analysis of the data-streams, which in turn, leads to the high-dimensionality challenge. To overcome this challenge, a desired anomaly detection technique needs to be scalable to high-dimensional data in real-time.

Anomaly detection in many systems such as fraud detection could be the ultimate goal, however, in many scenarios, such as diagnosis systems (e.g., spacecraft monitoring system [9]) and cybersecurity, it is highly important to provide a degree of interpretation about the detected issue in the system and how to mitigate it. Considering the potential damages caused by failure in mitigation of unexpected behaviors, such as cyber-attacks, detecting anomalies without providing any further information explaining where the anomaly has happened is of limited value to the engineers. Motivated by the aforementioned challenges, we investigate an *online multivariate anomaly detection and localization* technique which is simple enough to handle high-dimensional and heterogeneous data in real-time.

***Contributions:*** In this paper, aiming at the timely and accurate detection of anomalies in high-dimensional systems we propose a *k*NN-based sequential anomaly detection method. In summary, our contributions in this paper are as follows:

- A framework for multivariate, data-driven and sequential detection and localization of anomalies in high-dimensional systems is proposed for a semi-supervised setting where only a training data of nominal observations are available.
- Asymptotic optimality of the proposed detection method in the minimax sense is shown, and comprehensive analysis for computational complexity is provided.
- The practicality of the proposed anomaly detection and localization is demonstrated on variety of applications such as detection of IoT botnets, seizure onsets, and anomalous behavior in video surveillance, using synthetic and real data.

The rest of the paper is organized as follows. In Section 3, the mathematical formulation of the anomaly detection problem and the relevant background information are provided. The proposed anomaly detection is presented in Section 4. Specifically, the asymptotic optimality and computational complexity of the proposed detector are analyzed. Moreover, a fast and approximate variant is presented and evaluated. The proposed anomaly localization technique is provided in Section 5. In Section 6, we evaluate the proposed detector and localization techniques using synthetic and real data, such as seizure onset detection in brain, IoT botnet detection, and detection of anomalous behavior in video surveillance. Finally, we conclude the paper in Section 7.

## 2. Related Work

The problem of anomaly detection has been an important subject of study in several research communities, such as statistics, signal processing, machine learning, information theory, and data mining, either specifically for an application domain or as a generic method. To name a few, an SVM classification approach for anomaly detection was proposed in [10]; Bayesian methods were developed for social networks [11], partially observed traffic networks [12], and streaming environmental data [13]; deep neural network models were proposed for detecting anomalies multivariate time series [14,15,16,17,18]; several information theoretic measures were proposed in [19] for the intrusion detection problem; and two new information metrics for DDoS attack detection was introduced in [3]. Due to the challenging nature of the problem and considering the challenges posed by today’s technological advances such as big data problems, there is still a need for studying the anomaly detection problem.

Sequential anomaly detection techniques, compared to the outlier detection techniques [1], take also the history of observations into account rather than only the new observations. Sequential techniques are more suitable for real-time systems where timely and accurate detection of anomalies is important. The Cumulative Sum (CUSUM) detector [20] is a well-known sequential change detection technique that assumes probabilistic models for nominal and anomalous data points, and computes the cumulative log-likelihood-ratio (LLR) over time, declaring anomaly if the statistic exceeds a predefined threshold. The accuracy of assumed probabilistic models as well as the estimated parameters are the key factors in the performance of CUSUM and in general parametric methods. CUSUM is minimax optimum under the condition that the probability distributions before and after the change are completely known [21]. However, in many real-world applications having a complete *a priori* knowledge about the underlying distributions is not possible. Estimating the probability distributions quickly becomes intractable for high-dimensional data, which includes many unknowns, such as the anomaly onset time and subset of anomalous dimensions, in addition to the parameters of the nominal and anomalous models. To tackle with this complexity, ref.  [22] proposed a relaxed version of CUSUM in which each data stream is assumed to be independent of others. However, this univariate method is not suitable for detecting changes in the correlation between data streams. A sequential test for detecting changes in the correlation between variables, as well as localizing the highly correlated variables, in high-dimensional data streams has been proposed in [23]. This is a parametric method based on the assumption that the observed vectors are multivariate Gaussian distributed. It is proposed solely for the detection of correlation change between data streams and does not generalize to other changes in the distribution. In this paper, we are interested in detecting general changes in unknown distributions, including the changes in correlation structure.

*k*-nearest-neighbor (*k*NN) distance-based methods are geometric methods that are based on the assumption that anomalous data instances occur far from the nominal instances. For instance, refs. [24,25] have proposed nonparametric outlier detection techniques based on the minimum volume set (MVS) of the nominal data. MVS corresponds to the region of greatest probability density with minimum data volume and is known to be useful for anomaly detection [26] based on the assumption that anomalies occur in the less concentrated regions of the nominal dataset. These nonparametric outlier detection methods estimate the MVS of nominal training samples using *k*NN graphs, and declare a data point as anomalous if it lies outside the MVS. Despite being scalable to high-dimensional and heterogeneous data, they do not consider the temporal anomaly information, and thus are prone to higher false alarm rates compared to sequential anomaly detection methods. Similarly, ref. [27] proposed a *k*NN graph-based method that computes an anomaly score for each observation and declares an anomaly by thresholding the score value. In this paper, as opposed to the outlier detection methods which treat a single outlier as an anomaly, we consider an anomaly to consist of persistent outliers and investigate sequential and nonparametric detection of such anomalies using the temporal information in data streams. Recently, ref. [28] proposed a nonparametric *k*NN-based sequential anomaly detection method for multivariate observations. This method computes the test statistic based on the number of *k*NN edges at different splitting points within a window and stops the test whenever the test statistics exceed a threshold. Due to its window-based nature this method has inherent limitations in achieving small detection delays. It also recomputes the *k*NN graphs at every time instance and for every splitting point, therefore its computational complexity is note suitable for real-time applications. In another recent work,  ref. [29] proposed a distance-based and CUSUM-like change detection method for attributed graphs. Attributed graphs are first mapped into numeric vectors, and then the distance between the mean response of an observation window and the mean response of the training data are computed via a CUSUM-like sequential algorithm. In addition to the limitations arising from the window-based nature of the method, the local relations between samples are disregarded due to considering only the mean response of the training set. As a result, in cases where training data has a multimodal distribution, this method will not be effective. As compared to [29], we take into account the local relations between the data instances.

## 3. Problem Formulation

Throughout the paper, vectors and matrices are represented by boldface lowercase and uppercase letters, respectively. Script letters denote sets, e.g., X. Vectors are organized in a column unless otherwise stated. Probability and expectation are denoted with P and E, respectively. Suppose that a system is observed through *d*-dimensional observations $X_{t}=\left\{x_{1},x_{2},\ldots ,x_{t}\right\}$ in time. The objective is to detect an anomaly occurring at an unknown time τ as soon as possible while satisfying a false alarm constraint. This problem can be formulated as a change detection problem as follows:

(1) $$\begin{array}{cc} \; & f=f_{0},\;t<\tau ,\;f=f_{1}\left(\neq f_{0}\right),\;t\geq \tau , \end{array}$$

where *f* is the true probability distribution of observations, $f_{0}$ and $f_{1}$ are the nominal and anomaly probability distributions, respectively. The objective of the problem is to minimize the average detection delay while satisfying a false alarm constraint, i.e.,

(2) $$\underset{T}{\inf}E_{\tau }\left[{\left(T-\tau \right)}^{+}\right]\;\;\mathrm{subject}\;\mathrm{to}\;\;E_{\infty }\left[T\right]\geq \beta ,$$

where *T* denotes the alarm (i.e., stopping) time, $E_{\tau }$ represents the expectation given that change occurs at τ, ${\left(.\right)}^{+}=\max\left(.,0\right)$, and $E_{\infty }$ denotes the expectation given that no change occurs, i.e., the expectation of false alarm period.

Lorden’s minimax problem is a commonly used version of the above problem [30], in which the goal is to minimize the worst-case average detection delay subject to a false alarm constraint:

(3) $$\underset{T}{\inf}\underset{\tau }{\sup}\mathrm{ess}\underset{X_{\tau }}{\sup}E_{\tau }\left[{\left(T-\tau \right)}^{+}|X_{\tau }\right]\;\;s.t.\;\;E_{\infty }\left[T\right]\geq \beta ,$$

where “ess sup” denotes essential supremum which is equivalent to supremum in practice. In simple words, the minimax criterion minimizes the average detection delay for the least favorable change-point and the least favorable history of measurements up to the change-point while the average false alarm period is lower bounded by β.

The CUSUM test provides the optimum solution to the minimax problem [21], given by (3):

(4) $$\begin{array}{c} \begin{array}{cc} \; & S_{t}=\max\left\{0,S_{t-1}+\ell_{t}\right\},\\ \; & T_{c}=\inf\left\{t:S_{t}\geq h_{c}\right\}, \end{array} \end{array}$$

where $S_{t}$ is the cumulative decision statistic up to time *t* with $S_{0}=0$, $T_{c}$ is the stopping time, $\ell_{t}=\log\frac{f_{1}\left(x_{t}\right)}{f_{0}\left(x_{t}\right)}$ is the log-likelihood ratio at time *t*, and $h_{c}$ is a decision threshold, selected in a way to satisfy a given false alarm constraint. Considering $\ell_{t}$ as a statistical evidence for anomaly the CUSUM algorithm continues accumulating it, and declares an anomaly the first time the accumulated evidence $S_{t}$ exceeds a threshold $h_{c}$, that is chosen sufficiently large for reliable detection. CUSUM requires the complete knowledge of the probability distributions $f_{0}$ and $f_{1}$. However, in real-world applications, the true probability distributions are typically unknown. Even when $f_{0}$ and $f_{1}$ are known up to their parameters, and the parameters are estimated using the maximum likelihood approach, the procedure known as Generalized CUSUM (G-CUSUM) achieves only asymptotic optimality. Moreover, CUSUM and in general parametric methods are limited to the detection of certain anomaly types whose true probability distribution matches the assumed $f_{1}$ well.

In high-dimensional problems that require multivariate analysis, estimating the nominal probability distribution is typically not tractable, especially when the data dimensions are heterogeneous, e.g., environmental sensor data consisting of wind speed, direction, air temperature, pressure, humidity, weather condition (whether it is rainy, sunny or cloudy). Considering the wide range of possible anomalies it is even more intractable to estimate the anomaly probability distribution. In such problems, knowing the probability distributions and parameters is highly complicated if not impossible, limiting the applicability of CUSUM and parametric methods in general.

## 4. Proposed Detection Method

We propose a *k*NN-based sequential anomaly detection method called Online Discrepancy Test (ODIT). In this section, we elaborate on the motivation behind ODIT, prove its asymptotic optimality in the minimax sense under certain conditions, and extensively analyze its computational complexity.

**Figure 1:**
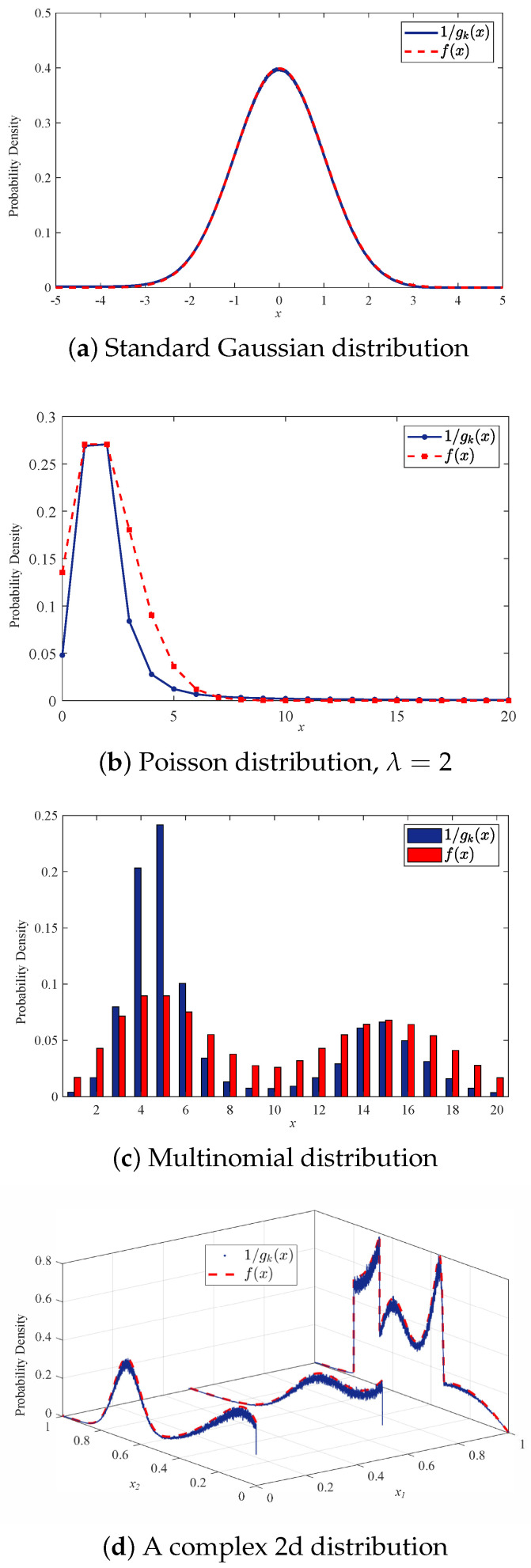
Similarity between inverse *k*NN distance $1/g_{k}\left(x\right)$ and likelihood f(x) for k=1.

The rationale behind using *k*NN distance for anomaly detection is the similarity between the inverse *k*NN distance and likelihood. Specifically, for $f\left(x_{i}\right)\geq f\left(x_{j}\right),\;x_{i},x_{j}\in X$, it is expected that the distance $g_{k}\left(x_{i}\right)$ of $x_{i}$ to its *k*th nearest neighbor in X is smaller than that of $x_{j}$. This probability increases with the size of X, i.e., $\lim_{|X|\to \infty }P\left(g_{k}\left(x_{i}\right)\leq g_{k}\left(x_{j}\right)\right)=1$. This in turn provides grounds for using the difference of *k*NN distances in ODIT to approximate the log-likelihood ratio $\ell_{t}$. The similarity between the likelihood of data points and the inverse *k*NN distance is shown in Figure 1 for several distributions. We consider Gaussian, Poisson and multinomial distributions to illustrate the similarity of $1/g_{k}\left(x\right)$ and f(x) for three disparate data types, real-valued numeric, integer-valued numeric and categorical, respectively. The inverse *k*NN distance graphs are scaled down to match the likelihood figure for the purpose of visualization. As shown in Figure 1a with $\left|X\right|=10^{6}$, the inverse of *k*NN distance approximates the likelihood very well for the standard Gaussian random variable. Despite some discrepancy for the Poisson and multinomial cases due to the discreteness of these random variables, it may still serve well the purpose of approximating the log-likelihood ratio. For these discrete cases, to avoid zero *k*NN distance we consider much smaller number of data points, 10 and 50 for Poisson and multinomial, respectively. Figure 1b,c are obtained by averaging over $5\times 10^{5}$ and $10^{4}$ trials, respectively. In order to show the similarity for a more complex distribution, in Figure 1d we consider a two-dimensional vector of a categorical random variable and a real-valued random variable with arbitrary distribution and $10^{4}$ data points.

### 4.1. Online Discrepancy Test (ODIT)

The proposed ODIT online anomaly detector is composed of two phases: (1) first through an offline training phase, the algorithm trains on a training set of nominal historic observations, (2) an online testing phase, in which the algorithm tests the incoming observations, until it detects a change in observations, w.r.t. the nominal notion. In the training phase, assuming a training set $X_{N}$ consisting of *N* nominal data instances, it randomly partitions $X_{N}$ into two sets $X_{N_{1}}$ and $X_{N_{2}}$, where $N_{1}+N_{2}=N$, for computational efficiency as in the bipartite GEM algorithm [25]. Then, using the *k*NN distances $\left\{g_{k}\left(x_{m}\right)\right\}$ between each node $x_{m}\in X_{N_{1}}$ and its *k* nearest neighbors in $X_{N_{2}}$ ODIT finds an estimate ${\hat{\Omega }}_{\alpha }$ for the minimum volume set (MVS) $\Omega_{\alpha }$ given by

(5) $$\Omega_{\alpha }=\underset{A}{\mathrm{argmin}}\int_{A}dx\;\;s.t.\;\;\int_{A}f_{0}\left(x\right)dx\geq 1-\alpha ,$$

where α∈(0,1) is a significance level, e.g., 0.05. $\Omega_{\alpha }$ represents the most compact set of observations under nominal operation while its complement ${\bar{\Omega }}_{\alpha }$ corresponds to the tail events (i.e., outliers) under nominal operation at significance level α. Then, in the test phase, it compares the *k*NN distances $g_{k}\left(x\right)$ between a test data instance x and its *k* nearest neighbors in $X_{2}$ with ${\hat{\Omega }}_{\alpha }$ to compute a negative/positive anomaly evidence for anomaly x and accumulates it over time for reliable detection. Roughly, the greater $g_{k}\left(x\right)$ is, the less likely x comes from the same distribution $f_{0}$ as the nominal points. The estimate ${\hat{\Omega }}_{\alpha }$ provides a reference to evaluate $g_{k}\left(x\right)$ and compute the negative/positive anomaly evidence for x.

Specifically, in the training phase, to estimate $\Omega_{\alpha }$ ODIT ranks the points in $X_{N_{1}}$ in the ascending order $\left\{x_{\left(1\right)},\ldots ,x_{\left(N_{1}\right)}\right\}$ in terms of the total distance

(6) $$L_{m}=\sum_{n=k-s+1}^{k}g_{n}{\left(x_{m}\right)}^{\gamma },$$

where $g_{n}\left(x_{m}\right)$ is the Euclidean distance between point $x_{m}\in X_{N_{1}}$ and its *n*th nearest neighbor in $X_{N_{2}}$, s∈[1,k] is a fixed number introduced for convenience, and γ>0 is the weight. Next, it picks the first *K* points $X_{N_{1}}^{K}=\left\{x_{\left(1\right)},\ldots ,x_{\left(K\right)}\right\}\subset X_{N_{1}}$ with the smallest total distances $\left\{L_{\left(1\right)},\ldots ,L_{\left(K\right)}\right\}$ to estimate the MVS $\Omega_{\alpha }$, i.e., ${\hat{\Omega }}_{\alpha }=X_{N_{1}}^{K}$. It is known [25] that $X_{N_{1}}^{K}$ converges to $\Omega_{\alpha }$ as

$$\lim_{K,N_{1}\to \infty }K/N_{1}\to 1-\alpha .$$

Hence, *K* is chosen as $K=\lfloor N_{1}\left(1-\alpha \right)\rfloor$, where ⌊·⌋ is the floor operator.

In the test phase, for each data instance $x_{t}$, ODIT firstly computes the total distance $L_{t}$ with respect to the second training set $X_{N_{2}}$ as in (6). Then, it computes the anomaly evidence, which could be either positive or negative, by comparing $L_{t}$ with the MVS model found in the training phase through the borderline total distance $L_{\left(K\right)}$

(7) $$D_{t}=d\left(\log L_{t}-\log L_{\left(K\right)}\right),$$

where *d* is the number of data dimensions. Finally, it updates a detection statistic $\Delta_{t}$ which accumulates the anomaly evidence $D_{t}$ over time, and raises an anomaly alarm the first time $\Delta_{t}$ crosses a predefined threshold,

(8) $$\begin{array}{c} \begin{array}{cc} \Delta_{t} & =\max\left\{\Delta_{t-1}+D_{t},0\right\},\;\Delta_{0}=0,\\ T & =\min\{t:\Delta_{t}\geq h\}, \end{array} \end{array}$$

which is a CUSUM-like procedure (cf. (4)). The ODIT procedure is summarized in Algorithm 1.
**Algorithm 1** The proposed ODIT procedure1:*Input:*$\;X_{N},k,s,\alpha ,h$2:*Initialize:* Δ←0,t←13:*Training phase:*4:Randomly partition $X_{N}$ into two sets $X_{N_{1}}$ and $X_{N_{2}}$5:For each $x_{m}\in X_{N_{1}}$ compute $L_{m}$ as in (6)6:Find $L_{\left(K\right)}$ by selecting the *K*th smallest $L_{m}$7:*Test phase:*8:**while** Δ<h **do**9:    Get new data $x_{t}$ and compute $D_{t}$ as in (7)10:    $\Delta =\max\{\Delta +D_{t},0\}$11:    t←t+112:**end while**13:DeclareAnomaly

The specific form of the anomaly evidence $D_{t}$ for each test instance $x_{t}$ enables the asymptotic optimality of ODIT in the minimax sense, as shown next.

**Theorem** **1.** *When the nominal distribution $f_{0}\left(x_{t}\right)$ is finite and continuous, and the anomalous distribution $f_{1}\left(x_{t}\right)$ is a uniform distribution, as the training set grows, the ODIT statistic $D_{t}$ converges in probability to the log-likelihood ratio,*

(9) $$D_{t}\overset{p}{\to }\log\frac{f_{1}\left(x_{t}\right)}{f_{0}\left(x_{t}\right)}\;\;\;\mathrm{as}\;\;\;N_{2}\to \infty ,$$

*i.e., ODIT converges to CUSUM, which is minimax optimum in minimizing expected detection delay while satisfying a false alarm constraint.*

**Proof.**  Consider a hypersphere $S_{t}\in R^{d}$ centered at $x_{t}$ with radius $g_{k}\left(x_{t}\right)$, the *k*NN distance of $x_{t}$ with respect to the training set $X_{N_{2}}$. The maximum likelihood estimate for the probability of a point being inside $S_{t}$ under $f_{0}$ is given by $k/N_{2}$. It is known that, as the total number of points grow, this binomial probability estimate converges to the true probability mass in $S_{t}$ in the mean square sense [31], i.e., $k/N_{2}\overset{L^{2}}{\to }\int_{S_{t}}f_{0}\left(x\right)\;dx$ as $N_{2}\to \infty$. Hence, the probability density estimate ${\hat{f}}_{0}\left(x_{t}\right)=\frac{k/N_{2}}{v_{d}g_{k}{\left(x_{t}\right)}^{d}}$, where $v_{d}g_{k}{\left(x_{t}\right)}^{d}$ is the volume of $S_{t}$ with the appropriate constant $v_{d}$, converges to the actual probability density function, ${\hat{f}}_{0}\left(x_{t}\right)\overset{p}{\to }f_{0}\left(x_{t}\right)$ as $N_{2}\to \infty$, since $S_{t}$ shrinks and $g_{k}\left(x_{t}\right)\to 0$. Similarly, considering a hypersphere $S_{\left(K\right)}\in R^{d}$ around $x_{\left(K\right)}$ which includes *k* points with its radius $g_{k}\left(x_{\left(K\right)}\right)$, we see that as $N_{2}\to \infty$, $g_{k}\left(x_{\left(K\right)}\right)\to 0$ and ${\hat{f}}_{0}\left(x_{\left(K\right)}\right)=\frac{k/N_{2}}{v_{d}g_{k}{\left(x_{\left(K\right)}\right)}^{d}}\overset{p}{\to }f_{0}\left(x_{\left(K\right)}\right)$. Assuming a uniform distribution $f_{1}\left(x\right)=f_{0}\left(x_{\left(K\right)}\right),\;\forall x$, we conclude with $\log\frac{\frac{k/N_{2}}{v_{d}g_{k}{\left(x_{\left(K\right)}\right)}^{d}}}{\frac{k/N_{2}}{v_{d}g_{k}{\left(x_{t}\right)}^{d}}}=d\left[\log g_{k}\left(x_{t}\right)-\log g_{k}\left(x_{\left(K\right)}\right)\right]\overset{p}{\to }\log\frac{f_{1}\left(x_{t}\right)}{f_{0}\left(x_{t}\right)}\;\;\mathrm{as}\;\;N_{2}\to \infty$, where $L_{t}=g_{k}\left(x_{t}\right)$ for s=γ=1. For γ values different than 1, $D_{t}$ converges to the log-likelihood ratio scaled by γ. □

Note that ODIT does not train on any anomalous data, i.e., does not use any knowledge of anomaly to be detected, while this generality is an attractive trait as it allows detection of any statistical anomaly, it also inevitably limits the performance for known anomaly types on which detectors can train. In Theorem 1, we show that in the lack of knowledge about anomalies, ODIT reasonably assumes an uninformative uniform likelihood for the anomaly case, and achieves asymptotic optimality under this assumption in the CUSUM-sense for certain parameter choices. However, ODIT is still effective when the anomalies do not follow the uniform distribution. The assumption of uniformly distributed anomalies is only needed for asymptotic optimality. In the experiments with real data presented in Section 6, where the anomalies follow non-uniform distributions, ODIT significantly outperforms the state-of-the-art methods.

*Remark 1 (Parameter Selection):* Due to its sequential nature, the parameters of ODIT either directly or indirectly control the fundamental trade-off between minimizing average detection delay and false alarm rate. Although ODIT has several parameters, the only main parameter that directly affects this trade-off is the detection threshold *h* in (8). Decreasing *h* will yield smaller detection delays, i.e., earlier detection, but also more frequent false alarms. As in other anomaly detection algorithms, it is typically selected to satisfy a false alarm constraint through a validation step. The other parameters of ODIT, $\alpha ,k,s,\gamma ,N_{1},N_{2}$ are all auxiliary variables introduced to increase the flexibility of ODIT. The performance of the proposed method is not very sensitive to the $\alpha ,k,s,\gamma ,N_{1},N_{2}$ values. For simplicity, they can be preset to fixed values without requiring any optimization through a validation process. For example, $k=s=\gamma =1,\alpha =0.05,N_{1}=N-N_{2}=0.3N$ are some typical values that will yield successful results in many applications. To further improve the performance of ODIT, these values can be optimized if desired. The significance level α is at a secondary role supporting *h*. For fixed *h*, larger α would result in a smaller estimated MVS ${\hat{\Omega }}_{\alpha }$, which in turn results in smaller detection delays, but also more frequent false alarms since more nominal data points will lie outside the selected MVS. Note that h is the final decision threshold, whereas α is more of an intermediate parameter. Hence, one can always set α to a reasonable significance value, such as 0.05, and then adjust *h* accordingly to satisfy a desired false alarm rate. Parameters *k* and *s* determine how many nearest neighbors to take into account in computing the total distance $L_{m}$, given by (6). Smaller *k* would result in being more sensitive to anomaly, hence supports earlier detection, but at the same time it causes to be more prone to the false alarms due to nominal outliers. Larger *k* would result in vice versa. *s* is an auxiliary parameter chosen for further flexibility in this trade-off. s=1 considers only the *k*th nearest neighbor while s=k sums all the first *k* nearest neighbors. Similar to *k*, smaller *s* makes the algorithm more sensitive to anomaly, but also more prone to nominal outliers. However, the effect of *s* is secondary to that of *k*. *k* and *s* should be chosen together to strike a balance between sensitivity to anomalies and robustness to nominal outliers. 0<γ<d is the weight which determines the emphasis on the difference between distances. Large distance values are emphasized by large γ values and suppressed by small γ values. Regarding the sizes of training sets $N_{2}$ plays a more important role than $N_{1}$, as shown in Theorem 1. Specifically, $N_{2}$ determines the accuracy of likelihood estimates by the *k*NN distances, whereas $N_{1}$ determines how well the significance level α is satisfied, which is an intermediate parameter as discussed before. Hence, typically $N_{2}$ should be chosen larger than $N_{1}$, where $N_{1}+N_{2}=N$. It should be noted that the ODIT procedure, given by Algorithm 1, can also work without partitioning the training set. Partitioning is proposed for computational efficiency when dealing with large high-dimensional datasets. However, it does not decrease the order of magnitude in computational complexity since even without partitioning the online testing procedure already scales linearly with the number of training instances, as opposed to the bipartite GEM algorithm [25] which decreases the complexity to linear from exponential using partitioning. As a result, Algorithm 1 can be used without partitioning the training set, especially for small datasets.

*Remark 2 (Graph Interpretation):* The *K* points in MVS estimate $X_{N_{1}}^{K}$ and their *k* nearest neighbors in $X_{N_{2}}$ form an Euclidean *k*NN graph $G=({\bar{X}}_{N_{1}}^{K},E)$, where ${\bar{X}}_{N_{1}}^{K}$ is the set of vertices and E is the set of edges connecting $X_{N_{1}}^{K}$ to the neighbors in $X_{N_{2}}$. The constructed graph G minimizes the total edge length $\sum_{m=1}^{K}L_{m}$ among all possible *K*-point *k*NN graphs between $X_{N_{1}}$ and $X_{N_{2}}$. The computation of anomaly evidence $D_{t}$ in (7) can then be interpreted as the increase/decrease in the log of total edge length if the *K*-*k*NN graph were to include the test point $x_{t}$.

*Remark 3 (Comparisons):* ODIT learns ${\hat{\Omega }}_{\alpha }$ using *k*NN distances similarly to the outlier detection method called Geometric Entropy Minimization (GEM) [24,25]. However, in the test phase, unlike GEM, which declares anomaly even when a single test point falls outside the MVS, ODIT sequentially updates a test statistic $\Delta_{t}$ using the closeness/remoteness of the test point to the MVS, and declares anomaly only when $\Delta_{t}$ is large enough, i.e., there is enough anomaly evidence with respect to a false alarm constraint. Doing so ODIT is able to timely and accurately detect persistent anomalies, as shown theoretically in Theorem 1, whereas one-shot outlier detectors like GEM are prone to high false alarm rates due to the limitation of significance tests [32,33]. The sequential detection structure of ODIT resembles that of CUSUM albeit with fundamental differences. Actually, the test statistic of ODIT implements a discrepancy function motivated by the discrepancy theory [34] and discrepancy norm [35], hence the name *Online Discrepancy Test (ODIT)*. The nonparametric nature of ODIT does not require any knowledge of the nominal and anomaly probability distributions, as opposed to CUSUM. Moreover, the practical relaxations of CUSUM, such as G-CUSUM and independent CUSUM [22], cannot be applied to challenging scenarios such as high-dimensional systems which require multivariate anomaly detection with little or no knowledge of anomaly types. On the other hand, ODIT scales well to high-dimensional systems for multivariate detection, as discussed next.

### 4.2. Computational Complexity

Next, we analyze the computational complexity of our proposed method. Training phase of ODIT requires the *k*NN distances between each pair of the data points in the two training sets. Therefore, the time complexity of training phase is $O(N_{1}N_{2}d)$, where *d* is the data dimensionality. The space complexity of training is $O(N_{2}d)$ since $N_{2}$ points are stored for testing. Note that training is performed once offline, thus the complexity of online testing is usually critical for scalability. In the test phase, computing the *k*NN distance of a test point among all points in the second training set takes $O(N_{2}d)$ time. The space complexity of testing is not significant as the test statistic is updated recursively. Consequently, the proposed ODIT algorithm linearly scales with the data dimensionality *d* both in training and testing. In the online testing phase, it also scales linearly with the number of training points. For high-dimensional systems with abundance of training data, the online testing time could be the bottleneck in implementing ODIT.

*kNN Approximation:* Computing the nearest neighbors of a query point is the most computationally expensive part of the algorithm as the distance to every other point in the second training data needs to be computed to select the *k* smallest ones. As the dimensionality increases and the training size grows, the algorithm becomes less efficient in terms of the running time. To this end, we propose to approximate the *k*NN distance rather than computing its exact value. It is natural to expect that ODIT’s performance will drop due to the inaccuracy induced by the approximated *k*NN distances compared to that based on the exact *k*NN distances. However, depending on the system specifications, e.g., how frequently the data arrives and how critical timely detection is, the reduction in running time through *k*NN approximation may compensate for the performance loss, as we next analyze through an experiment. Ref. [36] proposes a *k*NN distance approximation algorithm that scales well to high-dimensional data. This algorithm performs hierarchical clustering by constructing a k-means tree, and approximates the *k*NN distance by performing a priority search in the k-means tree, i.e., by searching for the *k* nearest neighbors only among a limited number of data points. The computation complexity of constructing the tree is $O(N_{2}dCI_{max}\frac{\log N_{2}}{\log C})$, where $I_{max}$ is the maximum number of iterations in k-means clustering, *C* is the number of clusters (a.k.a. branching factor), and $\frac{\log N_{2}}{\log C}$ is the average height of the tree. Using the priority search k-means tree algorithm, the computational complexity of *k*NN search reduces to $O(Bd\frac{\log N_{2}}{\log C})$, where $B\ll N_{2}$ is the maximum number of data points to examine. Hence, the training complexity reduces to $O(\left(N_{1}B+N_{2}CI_{max}\right)\frac{\log N_{2}}{\log C}d)$ from $O(N_{1}N_{2}d)$. Note that $B\ll N_{2}$ and the number of iterations required for convergence is small [36]. More importantly, in online testing, the computational complexity per instance decreases to $O(B\frac{\log N_{2}}{\log C}d)$ from $O(N_{2}d)$.

*Experiment:* We experimented with this approximation in our algorithm. The experiment is done in Matlab on an Intel 3.60 GHz processor with 32 GB RAM. In the experiment, the dimensionality of data is d=50, the training data size is $N=5\times 10^{5}$, partitioned into $N_{1}=0.38N$ and $N_{2}=0.62N$, and the anomaly is defined as a shift in the mean of Gaussian observations by 3 standard deviation in 10% of the dimensions. We set the branching factor for building the priority search k-means tree as C=100, and the maximum number of points to examine during search for the *k* nearest neighbors as B=1000. The average computation time for both ODITs based on the exact and the approximate *k*NN distance is summarized in Table 1, which presents the time spent for the computation of (7) and (8) per observation. It is seen that the approximation method drops the average running time per observation to about 1/14 of that of the exact method.

**Figure 2:**
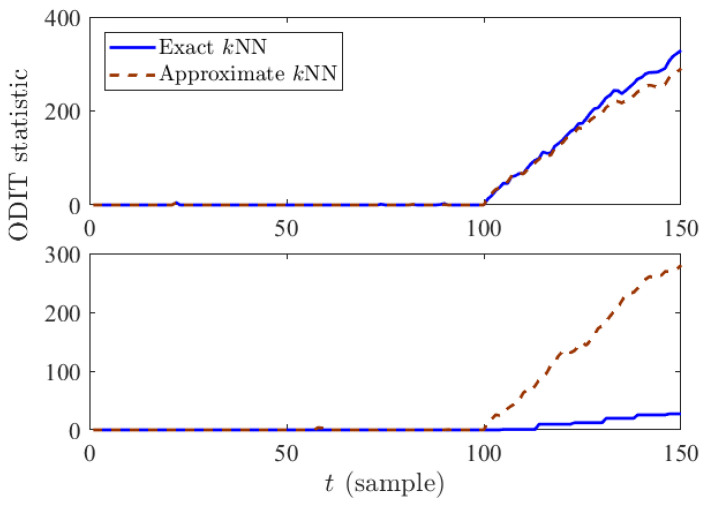
ODIT statistics based on exact and approximate *k*NN distances when $T_{\mathrm{sampling}}=1$ s (**top**) and $T_{\mathrm{sampling}}=0.01$ s (**bottom**).

**Figure 3:**
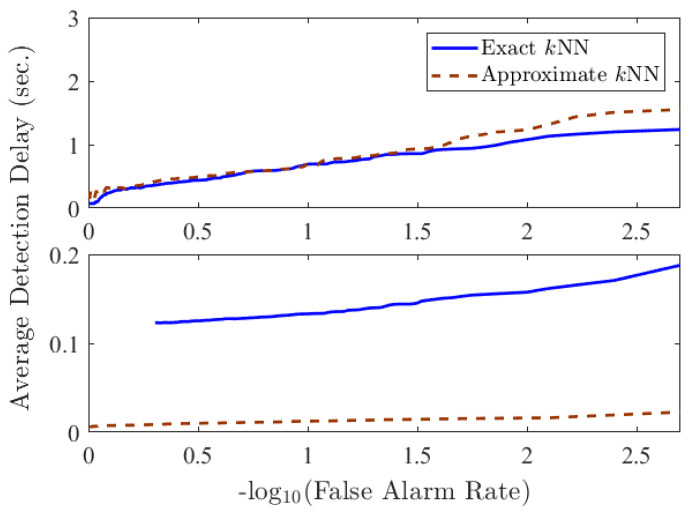
Comparison between performance of ODIT based on exact and approximate *k*NN distances in terms of seconds for $T_{\mathrm{sampling}}=1$ s (**top**) and $T_{\mathrm{sampling}}=0.01$ s (**bottom**).

To compare the original and efficient ODITs in systems with different specifications, in terms of the frequency of data arrival, we considered the following two scenarios: (i) data arrives every 1 s, and (ii) data arrives every 0.01 s. Figure 2 and Figure 3 compare the decision statistic $\Delta_{t}$ given in Equation (8) and average performance of ODIT based on exact and approximate *k*NN in the two scenarios. Considering the extra samples needed for detection after the anomaly onset, as well as the computation time overhead for the last sample before detection, the actual detection delay in time unit is given by sampledelay×samplingperiod+computationaloverhead. Depending on the sampling period, either exact *k*NN or approximate *k*NN could be more advantageous. For a sampling period that is smaller than the computation overhead, exact *k*NN computations are usually not feasible, causing the original ODIT to miss multiple samples while performing the test for a data instance, as can be seen in the staircase statistic in solid blue in the bottom figure of Figure 2. Therefore, in such a case, approximate *k*NN computations are preferred over the exact *k*NN computations in terms of the actual detection delay (see the bottom figure in Figure 3). Whereas for a sufficiently large sampling period, the delay is mainly due to the extra samples, thus exact *k*NN computations yield better results this case, as shown in the top figure in Figure 3.

*Summary of ODIT:* Here we highlight the prominent features of the proposed ODIT anomaly detector:

- The *sequential* nature of ODIT makes it suitable for *real-time* systems, and especially for systems in which *quick and accurate* detection is critical. Additionally, as the nominal training set grows, it asymptotically achieves the minimax optimality in terms of quick and accurate detection when anomaly is from uniform distribution.
- It is capable of performing *multivariate* detection in *high-dimensional* systems, as illustrated in Section 6, thanks to its *nonparametric* and *scalable* nature.
- ODIT can detect *unknown, rare, and previously unseen anomaly* types since it does not depend on any assumption about anomalies.

## 5. Anomaly Localization Using ODIT

In this section, we propose a localization strategy to identify the data dimensions in which the detected anomaly occurs so that necessary steps can be taken to mitigate the anomaly. Specifically, after an anomaly is detected in ODIT, our objective is to identify the dimensions that caused the detection statistic $\Delta_{t}$ to increase considerably, ultimately resulting in the detection. Our approach to perform this task is by examining the contribution of each dimension individually to the decision statistics. In the case of detection by ODIT, an increase in the total distance $L_{t}$, given by (6), leads to an increase in the anomaly evidence $D_{t}$, given by (7), finally leading to an increase in the detection statistic $\Delta_{t}$, given by (8), and consequently the anomaly alarm. Let us assume $x_{t}$ is the test data instance, and $\left\{y_{1},\ldots ,y_{k}\right\}$ are its *k* nearest neighbors in the train set. The total *k*NN distance $L_{t}=\sum_{n=k-s+1}^{k}{∥x_{t}-y_{n}∥}^{\gamma }$, for γ=2, can be written in terms of the *d* data dimensions as

(10) $$L_{t}=\sum_{i=1}^{d}\delta_{t}^{i},\;\mathrm{where}\;\delta_{t}^{i}=\sum_{n=k-s+1}^{k}{\left(x_{t}^{i}-y_{n}^{i}\right)}^{2},$$

and $x_{t}^{i}$ and $y_{n}^{i}$ are the *i*th dimensions of the observation $x_{t}$ and its *n*th nearest neighbor $y_{n}$. $\delta_{t}^{i}$ is the contribution of *i*th dimension of the observation $x_{t}$ at time *t* to the detection statistic. Therefore, by analyzing $\delta_{t}^{i}$ for each dimension *i* during the final increase period of $\Delta_{t}$, which causes the anomaly alarm, we can identify the dimensions in which anomaly has been observed. To this end, we propose to use a recent history of $Q_{i}=\left\{\delta_{q}^{i}:\;q=\hat{\tau }+1,\ldots ,\hat{\tau }+S,\;\forall i\right\}$ since the last time $\Delta_{q}=0$. This time $\hat{\tau }$, the most recent time instance when the detection statistic was zero, can be seen as an estimate of the anomaly onset time. Finally, we apply a *t*-test on the *S* samples in Q to decide whether each dimension *i* is anomalous.

In particular, we propose the following anomaly localization procedure after the alarm is raised at time *T*:

1. Find $\hat{\tau }=\max\left\{t<T:\;\Delta_{t}=0\right\}$
2. Compute the sample mean and sample standard deviation of $Q_{i}$ for each dimension *i*:
  (11) $$\begin{array}{cc} \; & {\bar{\delta }}_{i}=\frac{1}{S}\sum_{t=\hat{\tau }+1}^{\hat{\tau }+S}\delta_{t}^{i}\;\;\mathrm{and}\;\;\eta_{i}=\sqrt{\frac{1}{S-1}\sum_{t=\hat{\tau }+1}^{\hat{\tau }+S}{\left(\delta_{t}^{i}-{\bar{\delta }}_{i}\right)}^{2}} \end{array}$$
3. Identify the anomalous dimensions by applying a *t*-test:
  (12) $$\mathrm{if}\;\;\frac{{\bar{\delta }}_{i}-\mu_{i}}{\eta_{i}/\sqrt{S}}\geq \theta ,\;\;\mathrm{then}\;\mathrm{dimension}\;i\;\mathrm{is}\;\mathrm{anomalous},$$

where $\mu_{i}$ is the sample mean of nominal training $\left\{\delta_{1}^{i},\ldots ,\delta_{N_{1}}^{i}\right\}$ values, and θ is the (1−β)th percentile, for significance level β, of Student’s *t*-distribution with S−1 degrees of freedom.

The significance level β, for which a typical value is 0.05, controls a balance between sensitivity to anomalies and robustness to nominal outliers. For given β and *S* values, the threshold θ can be easily found from a lookup table for Student’s *t*-distribution (e.g., θ=6.314 for β=0.05 and S=2). The number of samples *S* needs to be at least 2 to have a degree of freedom at least 1. In practice, *t*-test is commonly used for small sample sizes, therefore *S* does not need to be large. Indeed, larger *S* would cause longer reaction time since the localization analysis would be performed at time $\hat{\tau }+S$, which could be greater than the detection time *T*, incurring extra delay for localization and reaction after detection.

## 6. Numerical Results

In this section, we evaluate the performance of our proposed algorithm using synthetic and real-world data, in a variety of applications. Anomaly localization is evaluated only when it is applicable to the data and application, i.e., if the anomaly (defined by the particular data and application) is manifested in a subset of the data dimensions and the ground truths of anomalous dimensions are available. We compare ODIT with the state-of-the-art methods suitable for each application using the performance metrics that are suitable to each problem and widely used in the literature.

### 6.1. Simulated Data: Change in the Mean

**Figure 4:**
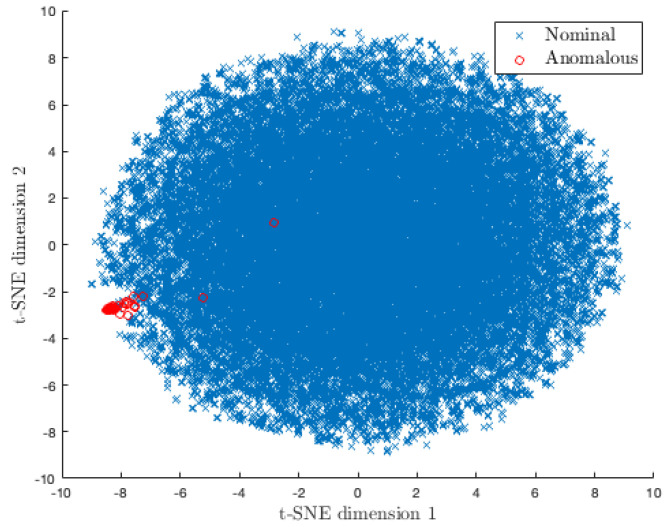
t-SNE plot [37] of the 50-dimensional nominal and anomalous data with change in the mean.

In this experiment, we compare and evaluate the detection and localization performance of ODIT with the benchmark methods on the simulated problem of detecting a small change in the mean of a multivariate distribution. We repeat the experiment discussed in Section 4.2 with the same simulation setup and ODIT parameters. The only difference is in the training data size, which is $N=5\times 10^{4}$. The anomalous data has a 3 standard deviation change in the mean of 5 attributes (10% of 50 dimensions). The nominal and anomalous data are visualized in Figure 4 using the t-SNE technique [37]. As seen in Figure 4, detecting anomalies in this scenario is a challenging task when the dimensionality is reduced to interpretable levels. We compare the performance of our proposed algorithm with CUSUM and two state-of-the-art change-point detection algorithms, namely sequential Nearest Neighbor (NN)-based CPD [28], and NEWMA [38]. The NN method is based on the two-sample test method proposed in [39,40], which, given two sample sets, determines whether they belong to the same distribution by employing a *k*NN similarity graph. NN-based sequential CPD performs two-sample test within a sliding window of observations, in a sequential manner moved by one instance at a time. The test stops as soon as the minimum normalized number of edges between two samples over all possible partitions is sufficiently low.

NEWMA [38] is an online and multivariate CPD algorithm, that is based on the Exponential Weighted Moving Average algorithm (EWMA). EWMA recursively computes a statistic with an exponential forgetting factor and raises an alarm if the statistics becomes too far apart from a known value (i.e., it requries a prior knowledge). NEWMA on the other hand does not require a prior knowledge and instead employs two different EWMA statistics with different forgetting factors. Rather than explicit selection of the two forgetting factor hyperparameters, authors propose selection of a window *L*, which represents the number of recent samples being compared to old samples. The forgetting factors are then selected depending on the choice of *L*. In our experiments, NN and NEWMA are evaluated using different window sizes.

**Figure 5:**
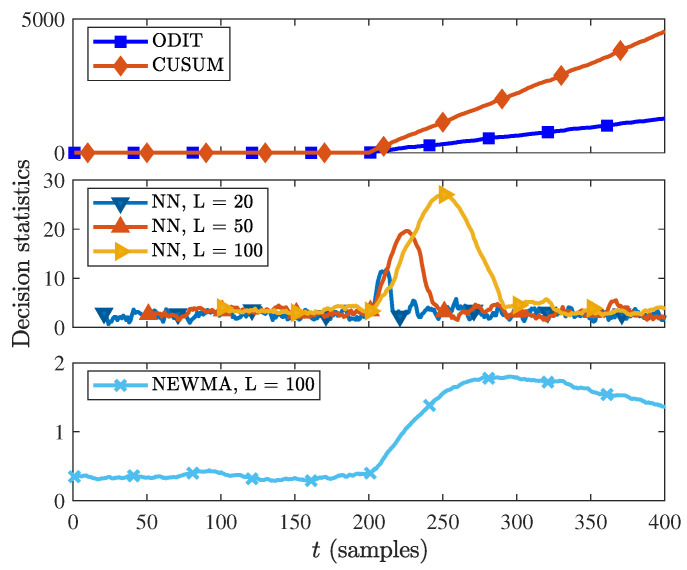
Decision statistics of ODIT, CUSUM, NN and NEWMA methods in the synthetic change-in-the-mean experiment.

The decision statistics of the four algorithms CUSUM, ODIT, NN and NEWMA are shown in Figure 5. The anomaly (change-point) occurs at time τ=200. As the observations after t≥τ are from a different probability distribution with respect to the nominal data, the ODIT and CUSUM statistics start increasing steadily after t=τ. On the other hand, the NN and NEWMA methods, which are both window-based methods, show increase in the decision statistics when the change point τ falls within the test observation window, and decrease after the window passes the change point. Since for smaller window sizes the increase in decision statistics is smaller, the NEWMA and NN algorithms can fail to detect the change point due to the small window size.

**Figure 6:**
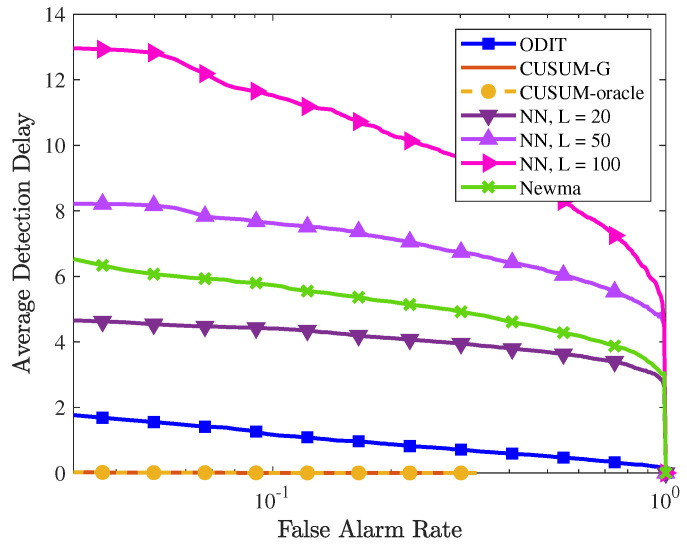
Performance comparison of the proposed ODIT, CUSUM, NN, and NEWMA detectors in the synthetic change-in-the-mean experiment in terms of average detection delay vs. FAR (in log-scale).

The performances of all detectors are compared in Figure 6. The performance of NN for different window sizes confirms that, as the window size increases, the change point is detected with a larger delay. In this experiment, all algorithms achieve 100% detection, that is, the change point is detected in all trials for all false alarm rates. In terms of average detection delay, CUSUM and generalized CUSUM (CUSUM-G) achieve almost zero detection delay since the assumed probability distribution (multivariate Gaussian) conforms with the true one. However, in real-world applications it is typically not possible to know the true pre- and post-change probability distributions, which consequently limits the applicability of CUSUM and CUSUM-G in real-world applications. The proposed ODIT detector achieves much smaller detection delay compared to the practical competitors (NN and NEWMA) from the literature. The window size L=100 gave the best performance for NEWMA.

**Figure 7:**
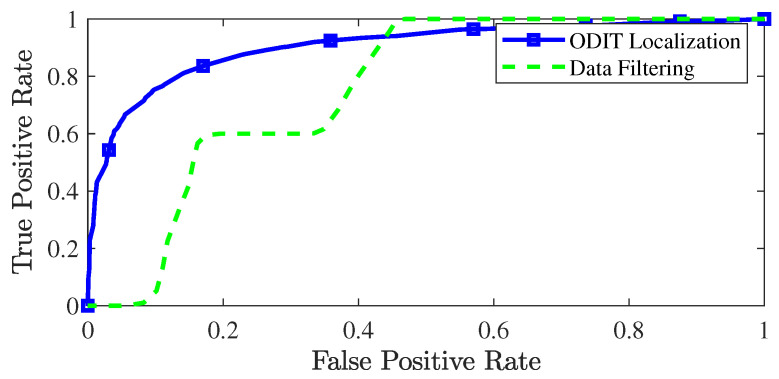
ROC curve of ODIT and the conventional data filtering approach for identifying anomalous dimensions in the change-in-the-mean experiment.

The ROC curve of ODIT for localization of the anomalous dimensions is shown in Figure 7 and compared with the straightforward data-filtering approach since the state-of-the-art detectors do not have a procedure for anomaly localization. The AUC for ODIT localization achieves 0.9041 while the detector satisfies the false alarm rate of 0.01. The conventional data filtering approach identifies a dimension as anomalous if its value exceeds a predefined threshold. Due to the small change ratio in the experiment, the data filtering approach fails to attain high identification probability while satisfying small false positive rates.

### 6.2. Simulated Data: Change in the Correlation

**Figure 8:**
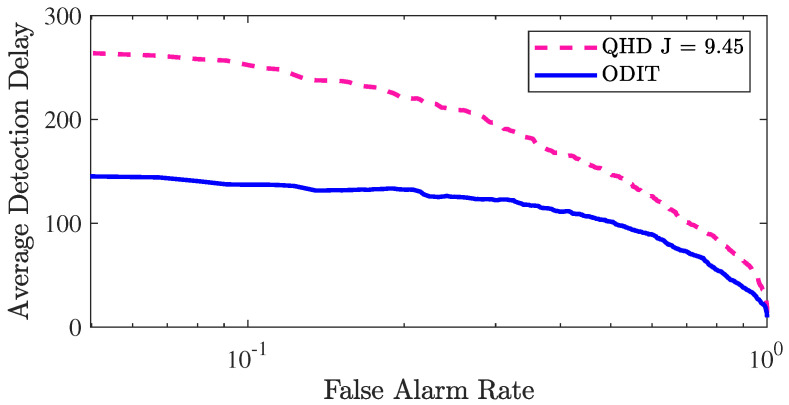
Performance evaluation for ODIT and QHD in the correlation monitoring example in terms of average detection delay vs. false alarm rate (log-scale).

The nonparametric nature of the proposed ODIT detector makes it suitable for multivariate detection in high-dimensional and heterogeneous systems. We next show the advantage of ODIT over a state-of-the-art correlation change detection method [7] in a challenging setting where anomaly is manifested as a change in the correlation structure between the individual data streams. The practical importance of this type of anomaly is well exemplified by the MadIoT attacks introduced in [8], in which high wattage IoT devices, such as air conditioners and water heaters, are synchronously turned on/off to cause instability and as a result blackout in the power grid. Following the experiment settings presented in [7] we simulate a 100-dimensional system that nominally generates data from a multivariate Gaussian distribution with diagonal covariance $\Sigma_{0}=\mathrm{diag}\left(\sigma_{i}^{2}\right)$, where $\sigma_{i}^{2}>0$ is the randomly chosen variance for each dimension. After the change point τ=100, 10 data streams become correlated. Specifically, the post-change covariance matrix $\Sigma_{1}$ is generated by replacing a random 10×10 block of $\Sigma_{0}$ with a random matrix sampled from the Wishart distribution. We make sure that the pre-change and post-change variances remain the same and only the correlations change. The size of the nominal training dataset is $N=10^{4}$. The detector proposed in [7] (QHD) is a nonparametric quickest change detection algorithm that focuses on detection of change in the correlation structure of data. Similar to ODIT, it aims to minimize the average detection delay while satisfying a false alarm constraint. The considered problem is significantly more challenging than the change-in-the-mean problem due to the fact that the mean and variance of individual data-streams do not change. In particular, data instances after the anomaly onset are still very similar to the nominal instances. To cope with the similarity of the anomaly instances to the nominal ones, the parameters of ODIT algorithms are set to be k=s=γ=1, α=0.1. Figure 8 compares the detection performances of ODIT and QHD. The *J* parameter of QHD is optimized as 9.45. As seen in the figure, especially in the low FAR regime, ODIT successfully minimizes the average detection delay compared to QHD, which specializes on correlation change detection.

### 6.3. Seizure Detection

**Figure 9:**
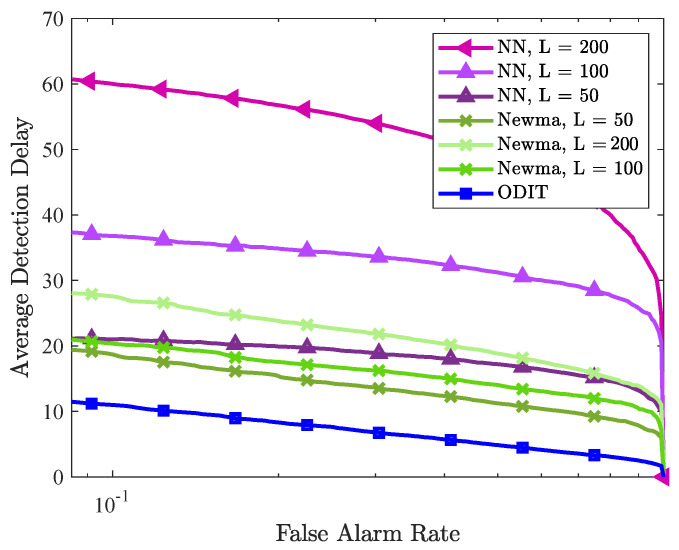
Performance comparison of ODIT, NN, NEWMA detectors in the seizure detection experiment. The performances are evaluated in terms of average detection delay vs. false alarm rate (in log-scale) and are averaged over all patients and dog subjects.

**Figure 10:**
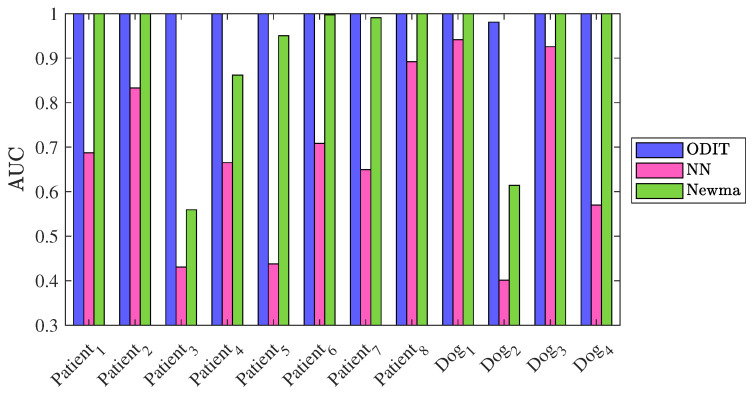
Performance comparison of the detectors in terms of the AUC of ROC curve for each subject.

Next, as a real-world application we consider the problem of detecting seizure in intracranial EEG signals. For patients unresponsive to the medication, the quick and accurate detection of seizure onsets are crucial for timely neurostimulation treatment to be effective and stop the seizure. Therefore, seizure detection is a suitable application to evaluate our proposed method. We use the dataset provided in a Kaggle competition, namely UPenn and Mayo Clinic’s seizure detection challenge. The dataset contains EEG signals of 12 subjects, 8 human patients, and 4 dogs. The dataset for each subject is provided in clips of duration 1 s. (with varying number of samples due to different sampling frequencies), where each clip belongs to either interictal (nominal) class or the ictal (seizure or anomaly) class. The dimensionality of signals for each subject varies due to different number of electrodes for signal sampling. The ground truth (i.e., nominal/seizure labels) are not available for the test data clips provided in the dataset. Therefore, we trained the algorithms on a portion of the training data, and tested them on the rest of the training data. For each subject, we trained ODIT on a nominal dataset of size *N* = 20,000 and arranged the testing data for each trial to contain 200 nominal samples followed by 200 anomalous samples. The algorithm parameters in this experiment are set to be k=s=γ=1, α=0.2. Figure 9 demonstrates the performance comparison of the methods in terms of average detection delay vs. false alarm rate, averaged over all the subjects. ODIT can detect seizure as early as 10 samples after the seizure onset when only 0.01 of the detections are false alarms. With the same window size, NEWMA achieves smaller average detection delay compared to NN, both of which achieve detection delay around 20 samples while satisfying 0.01 FAR for window size 50. As the window sizes of NEWMA and NN increases, the detection delay increases for both algorithms. ODIT achieves the lowest average detection delay, as well as the highest AUC for detection, as shown in Figure 10 (AUC is 1 for all subjects except for the subject Dog 2, for which the AUC value is 0.9809). According to Figure 10, NEWMA outperforms NN (both with window size L=50) in terms of AUC for each patient.

### 6.4. IoT Botnet Detection

In this section, experiments are performed on the N-BaIoT Botnet attack detection dataset, which consists of real IoT data traffic observations. These data are collected from 9 IoT devices infected by the Mirai and BASHLITE malware [41,42]. Here we only consider the Mirai attack dataset. The benign and attack datasets for each device is composed of 115 features summarizing traffic statistics over different temporal windows. The dataset is collected for each device separately and lacks timestamp. Therefore, we formed the training and test sets by randomly choosing data instances from each device. To form a network-wide instance for multivariate detection we stack the chosen instances from 9 devices into a single vector of 1035 dimensions. This way, we obtained a nominal train set with *N* = 10,000 instances. We form the test data similarly to the training data, assuming that a randomly selected device gets compromised and starts sending malicious traffic at time instance τ=101. For ODIT, we set parameters as k=s=γ=1, α=0.05.

**Figure 11:**
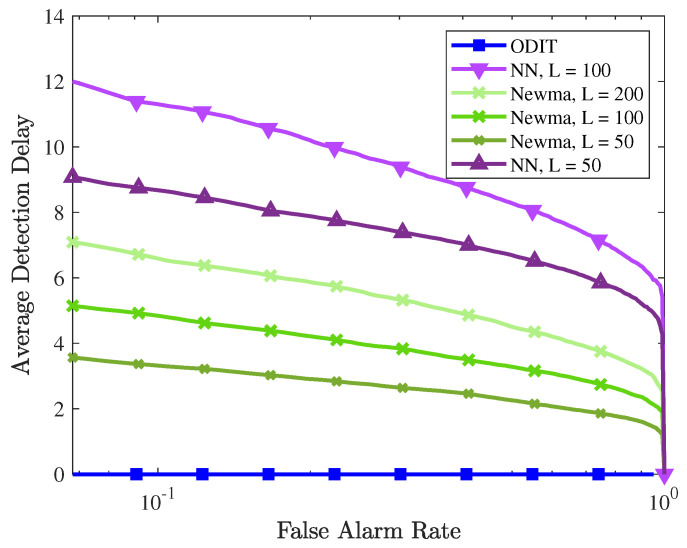
Performance evaluation of anomaly detectors in the botnet detection experiment in terms of the average detection delay vs. FAR.

In the experiments, ODIT detects the attack with a zero detection delay for all false alarm rates in all trials for any threshold value h>0. Performance of the three methods are compared in Figure 11 in terms of average detection delay vs. false alarm rate. It is evident that all methods, achieve low detection delays while ODIT achieves the smallest detection delay of 0 for all false alarm rate constraints. All three detectors successfully detect all attacks under all false alarm constraints, achieving the AUC value of 1. We also compare the performance of ODIT to the deep autoencoder-based detection method, proposed in the original N-BaIoT paper [41]. Autoencoder-based method trains the model and performs anomaly detection on each device separately. Hence, to compare methods, we also run ODIT on all devices separately (i.e., instead of performing network-wide anomaly detection, we perform anomaly detection for each device separately). Deep autoencoder is a deep neural network architecture composed of an encoder-decoder pair, which first maps the inputs to their compressed representations (encode) and reconstructs the original inputs from the representations (decode). Trained on the nominal data, the model is expected to have small and large errors, respectively, on reconstructing nominal and anomalous test data. By thresholding the model output for each observation, this method marks each observation instance as nominal or anomalous and employs majority voting on a moving window of size $ws^{*}$ (to control the false positive rate). It raises alarm only if the majority of the instances within the window are marked as anomalous. Due to its window-based majority rule, the sample detection delay (i.e., the number of anomalous instances observed before the detection) is at least $\lfloor \frac{ws^{*}}{2}\rfloor +1$. For instance, the false positive rate and average detection delay for devices 1-9, respectively, are (0.01,42), (0.012,11), (0.007,10), (0.024,33), (0.01,17), (0,22), (0,11), (0,12), (0,13). Whereas, the sequential nature of ODIT enables immediate detection together with zero false alarm. The optimum window sizes reported in [41] for each device are used for the autoencoder method.

### 6.5. Detection of Abnormal Behavior in Surveillance Videos

Automated detection of abnormal events in video surveillance is a time-critical and challenging problem. In this section, we evaluate our proposed method using benchmark video anomaly detection datasets, namely ShanghaiTech [43], CUHK Avenue [44], and UCSD Ped2 [45]. ShanghaiTech dataset consists of 330 training videos and 107 test videos, recorded in 13 different scenes within the campus of ShanghaiTech University. CUHK Avenue dataset includes 16 training videos and 21 test videos, where the abnormal behavior is defined as loitering, running, and throwing objects. UCSD Ped2 dataset consists of 16 training videos and 12 test videos, in which the presence of non-pedestrians is considered anomalous. For these experiments, we first extract informative features from video frames and then use ODIT on the extracted features. Our feature extraction component utilizes a Generative Adversarial Network (GAN) based future frame prediction [46], and an object detector (YOLOv3) [47] to extract motion, location, and appearance features to facilitate detection of a broad and unknown class of anomalous events. For each detected object *i* in a video frame $X_{t}$, we form a feature vector $F_{t}^{i}$, which consists of the mean squared error $MSE(X_{t},{\hat{X}}_{t})$ from the future frame prediction component, the center coordinates and area of the bounding box of the detected object, and the class probabilities of the detected object. After an anomaly is detected, for temporal localization of the anomaly, we perform some fine tuning to better label video frames as nominal or anomalous. Specifically, we find the frame ODIT statistic $\Delta_{t}$ started to grow, i.e., the last time $\Delta_{t}=0$ before detection, say $\tau_{\mathrm{start}}$. Then, we also determine the frame $\Delta_{t}$ stops increasing and keeps decreasing for n, e.g., 5, consecutive frames, say $\tau_{\mathrm{end}}$. Finally, we label the frames between $\tau_{\mathrm{start}}$ and $\tau_{\mathrm{end}}$ as anomalous, and continue testing for new anomalies with frame $\tau_{\mathrm{end}}+1$ by resetting $\Delta_{\tau_{\mathrm{end}}}=0$.

We compare our proposed method with several state-of-the-art deep learning-based video anomaly detection methods including MPPCA [48], MPPC + SFA [45], Del et al. [49], Conv-AE [50], ConvLSTM-AE [51], Growing Gas [52], Stacked RNN [43], Deep Generic [53], GANs [54], Sultani et al. [2], and Liu et al. [46]. Table 2 compares the performance of our proposed algorithm with those of the benchmark methods in terms of the frame-level AUC, the commonly used performance metric in video anomaly detection. Frame-level AUC is the area under the ROC (receiver operating characteristic) curve which plots true positive (alarm) rate vs. false positive (alarm) rate considering video frames as data instances. The AUC values in Table 2 are in the percentage format. It is seen that the proposed detector outperforms the benchmark algorithms with a significant margin in the CUHK Avenue and UCSD Ped2 datasets, and achieves a competitive performance in ShanghaiTech dataset. Since the proposed method uses commonly used feature extractors and differ from the existing methods in the detection technique, these results show the effectiveness of the proposed ODIT detector. In the case of the ShanghaiTech dataset, although [46] achieves a higher AUC, their decision methodology depends on the normalization of the computed statistics per each video, which requires that the entire video is seen before the anomalous frames are detected and thus prevents real-time detection. Unlike this method, ODIT is based on online decision making and can detect anomalies in streaming videos in real-time. In the experiments, the proposed video anomaly detector makes real-time decisions at the speed of 25 frames per second (fps). It should be noted that the GAN-based feature extraction is the limiting part in the 25 fps performance. Even in the largest dataset, ShanghaiTech, which has around 300,000 training frames and ten object types, ODIT processes more than 40 fps with exact kNN computations and around 560 fps with approximate kNN computations.

## 7. Conclusions

In this paper, we proposed an online and nonparametric anomaly detection algorithm, ODIT, that enables quick and accurate anomaly detection and localization in high dimensional systems that require multivariate (i.e., joint) monitoring of the system components. Our proposed anomaly detection method is generic and applicable to various contexts as it does not assume specific data types, probability distributions, and anomaly types. It only requires a nominal training set and achieves asymptotic optimality in terms of minimizing average detection delay for a given false alarm constraint. We evaluated the performance of our method in the context of seizure detection, botnet detection, and video anomaly detection using real datasets, as well as with synthetic data. The experiments verified the superior performance of the proposed method in online detection and localization of anomalies as compared to the state-of-the-art algorithms. Extending it to dynamic settings, such as an IoT network with dynamic topology and changing nominal behavior, remains to be an important future research direction.

## Figures and Tables

**Table 1:** Average computation overhead of original ODIT and efficient ODIT per sample.

Average Execution Time (s)	
Exact *k*NN	Approximate *k*NN
0.0750	0.0054

**Table 2:** Frame-level AUC comparison on three datasets with benchmark algorithms from the literature. AUC values are shown in the percentage format, where 100 is the highest possible score and 0 is the lowest.

Methodology	CUHK Avenue	UCSD Ped 2	ShanghaiTech
MPPCA [48]	-	69.3	-
MPPC + SFA [45]	-	61.3	-
Del et al. [49]	78.3	-	-
Conv-AE [50]	80.0	85.0	60.9
ConvLSTM-AE [51]	77.0	88.1	-
Growing Gas [52]	-	93.5	-
Stacked RNN [43]	81.7	92.2	68.0
Deep Generic [53]	-	92.2	-
GANs [55]	-	88.4	-
Liu et al. [46]	85.1	95.4	**72.8**
**Ours**	**86.4**	**97.2**	70.9

## Data Availability

Not applicable.
